# Derivation and internal validation of a multi-biomarker-based cardiovascular disease risk prediction score for rheumatoid arthritis patients

**DOI:** 10.1186/s13075-020-02355-0

**Published:** 2020-12-04

**Authors:** Jeffrey R. Curtis, Fenglong Xie, Cynthia S. Crowson, Eric H. Sasso, Elena Hitraya, Cheryl L. Chin, Richard D. Bamford, Rotem Ben-Shachar, Alexander Gutin, Darl D. Flake, Brent Mabey, Jerry S. Lanchbury

**Affiliations:** 1 University of Alabama at Birmingham, Birmingham, AL USA; 2 Mayo Clinic, Rochester, MN USA; 3 Crescendo Bioscience, South San Francisco, CA USA; 4 Myriad Genetics Laboratories, Salt Lake City, UT USA

**Keywords:** Biomarker, Cardiovascular risk, MBDA score, Multi-biomarker, Myocardial infarction, Rheumatoid arthritis, Stroke

## Abstract

**Background:**

Rheumatoid arthritis (RA) patients have increased risk for cardiovascular disease (CVD). Accurate CVD risk prediction could improve care for RA patients. Our goal is to develop and validate a biomarker-based model for predicting CVD risk in RA patients.

**Methods:**

Medicare claims data were linked to multi-biomarker disease activity (MBDA) test results to create an RA patient cohort with age ≥ 40 years that was split 2:1 for training and internal validation. Clinical and RA-related variables, MBDA score, and its 12 biomarkers were evaluated as predictors of a composite CVD outcome: myocardial infarction (MI), stroke, or fatal CVD within 3 years. Model building used Cox proportional hazard regression with backward elimination. The final MBDA-based CVD risk score was internally validated and compared to four clinical CVD risk prediction models.

**Results:**

30,751 RA patients (904 CVD events) were analyzed. Covariates in the final MBDA-based CVD risk score were age, diabetes, hypertension, tobacco use, history of CVD (excluding MI/stroke), MBDA score, leptin, MMP-3 and TNF-R1. In internal validation, the MBDA-based CVD risk score was a strong predictor of 3-year risk for a CVD event, with hazard ratio (95% CI) of 2.89 (2.46–3.41). The predicted 3-year CVD risk was low for 9.4% of patients, borderline for 10.2%, intermediate for 52.2%, and high for 28.2%.

Model fit was good, with mean predicted versus observed 3-year CVD risks of 4.5% versus 4.4%. The MBDA-based CVD risk score significantly improved risk discrimination by the likelihood ratio test, compared to four clinical models. The risk score also improved prediction, reclassifying 42% of patients versus the simplest clinical model (age + sex), with a net reclassification index (NRI) (95% CI) of 0.19 (0.10–0.27); and 28% of patients versus the most comprehensive clinical model (age + sex + diabetes + hypertension + tobacco use + history of CVD + CRP), with an NRI of 0.07 (0.001–0.13). C-index was 0.715 versus 0.661 to 0.696 for the four clinical models.

**Conclusion:**

A prognostic score has been developed to predict 3-year CVD risk for RA patients by using clinical data, three serum biomarkers and the MBDA score. In internal validation, it had good accuracy and outperformed clinical models with and without CRP. The MBDA-based CVD risk prediction score may improve RA patient care by offering a risk stratification tool that incorporates the effect of RA inflammation.

## Background

Cardiovascular disease (CVD) is the leading cause of mortality for patients with rheumatoid arthritis (RA), accounting for 30–40% of deaths [1]. Patients with RA have approximately 50% greater risk for cardiovascular disease (CVD) compared to the general population [2]. Traditional CVD risk factors such as diabetes, hypertension, and hyperlipidemia are important in RA patients and are not difficult to assess. However, the time constraints of a busy office practice often preclude making CVD risk stratification a routine part of RA patient care. Indeed, 79% of rheumatologists cite a lack of time as a major barrier [3]. Even so, rheumatologists are well positioned to help manage CVD risk in RA patients because 30% of CVD risk in RA patients is attributable to systemic inflammation and other RA-related factors [4, 5].

CVD risk predictors developed for the general population tend to underestimate CVD risk in RA patients [6–8]. European League Against Rheumatism (EULAR) guidelines recommend that CVD risk predicted by tools such as the Framingham Risk Score (FRS) or the American College of Cardiology and American Heart Association (ACC/AHA) pooled cohort risk equation [9] be multiplied by 1.5 to account for the effect of RA on CVD risk [6, 10]. A limitation of this approach is that it treats all RA patients the same, regardless of the level of disease activity.

ACC/AHA guidelines recommend preventive strategies for all patients with high predicted risk of CVD. Current recommendations support managing hyperlipidemia by “treating to risk” rather than a targeted LDL [11–13]. It is well established that vascular inflammation has a central role in atherosclerosis and CVD, but evidence that reducing systemic inflammation has potential to lower CVD risk is more recent. Proof of principle comes from the CANTOS trial, which showed that canakinumab, an anti-IL-1β biologic drug, reduced the CVD event rate in non-RA patients with a high risk of CVD and elevated high-sensitivity C-reactive protein (CRP) [14]. Patients with greater reduction in inflammation, measured by CRP, benefited the most [15].

Synovial and systemic inflammation in RA patients contribute to CVD risk independently of traditional risk factors [4]. In observational studies, the risk for CVD events was greatest in RA patients with high disease activity [16–20] and effective RA treatment appeared to reduce the risk for atherosclerosis [21] and CVD events [22, 23]. Traditional CVD risk factors, such as diabetes, may be exacerbated by RA-related mechanisms [24, 25]. Thus, it may be possible to reduce the CVD risk elevation attributable to RA by treating RA inflammatory pathways.

High sensitivity CRP has prognostic value for CVD events in non-RA populations, but its role for CVD risk prediction in RA patients is less clear because CRP may be a marker for systemic inflammation in RA rather than a surrogate for the extent of vascular involvement [26]. Moreover, CRP is not elevated in some RA patients with active disease [27]. CVD risk prediction models that combine measures of RA disease activity with traditional risk factors [19, 28, 29] are not yet the standard of care. Molecular markers of inflammation other than CRP have not been incorporated into validated CVD risk predictors for RA patients. Their inclusion would be novel and may have potential to improve CVD preventive care for RA patients by making CVD risk stratification more accurate and accessible.

The multi-biomarker disease activity (MBDA) test assesses RA disease activity by measuring 12 serum protein biomarkers to provide a validated score on a scale of 1–100 that correlates with the Disease Activity Score in 28 joints with CRP (DAS28-CRP) [30]. In 2019, the American College of Rheumatology disease activity measures working group concluded that the MBDA score was one of 11 measures of RA disease activity that met the minimum standard for regular use [31]. The MBDA score is predictive of future radiographic damage, independently of other measures [32, 33]. In a large, cross-sectional observational study, the MBDA score was found to be associated with risk for CVD, suggesting that the MBDA score and at least some of its biomarkers detect inflammation that is relevant to cardiovascular pathology [16].

Building on this evidence, we now describe the development and internal validation of an RA-specific CVD risk prediction score that uses routine clinical assessments plus RA-related biomarkers to predict CVD risk. The goal of this approach was to improve preventive CVD care in RA patients by developing a prognostic score that uses biomarkers to incorporate the contribution of RA-related inflammation to individual CVD risk. The intended end result of this endeavor is to create a validated CVD risk score that will enable rheumatologists to risk stratify their RA patients efficiently in an office setting, with components associated with RA disease activity directly represented in the CV risk estimate.

## Methods

### Data source

A retrospective RA cohort was created for this study by linking claims data in the Medicare database with data in the MBDA test commercial database (Vectra®, formerly Crescendo Bioscience, Inc., South San Francisco, CA, USA, currently Myriad Genetics Laboratories, Salt Lake City, UT, USA), using all fee-for-service Medicare data from 2006 to 2016 for all individuals who underwent MBDA testing. Data were linked on patient date of birth, sex, MBDA test date, MBDA testing codes (defined by Current Procedural Terminology codes 81479, 83520, 84999, 86140, and 81490, submitted by Crescendo Bioscience or Myriad Genetics Laboratories), and the National Provider Identifier of the treating rheumatologist. Data were linked deterministically, using established methods [16, 34]. The University of Alabama at Birmingham institutional review board approved the study.

### Participant and MBDA test eligibility criteria

The patient cohort and MBDA test results included in this study were selected by applying a series of criteria to the patients and MBDA tests in the linked database described above (Supplemental Table 1). To be eligible for inclusion in the study, patients were required to (1) be ≥ 40 years old, (2) have at least one RA diagnosis code from a rheumatologist (ICD9 714.0; ICD10 M05.*, M06.*, excluding M06.4 and M06.1, with * representing any number of digits or characters), (3) have received an RA-specific treatment (TNF-inhibitor, abatacept, rituximab, anti-IL-6R, Janus kinase inhibitor, conventional synthetic disease-modifying anti-rheumatic drug including methotrexate, sulfasalazine, leflunomide and hydroxychloroquine) anytime up to and including the date of the first MBDA test, and (4) have at least one linked MBDA test result. The accuracy of this claim-based method of identifying RA patients exceeds 85% [35] and is likely made greater here by the linkage with data from MBDA testing, which is only for patients diagnosed with RA.

The baseline period for a patient was defined as the interval preceding the date of the first MBDA test in the linked database. It included all available preceding Medicare data and was required to span at least 1 year, with patients being required to have had at least 365 days of continuous coverage with Medicare parts A (hospital coverage), B (outpatient coverage), and D (pharmacy coverage). Patients were excluded if they had any diagnosis code in the baseline period for malignancy (except non-melanoma skin cancer), myocardial infarction (MI), or stroke. MBDA test results (i.e., the MBDA score and 12 biomarker measurements) were used from the earliest MBDA test performed after the above requirements had been met, unless (1) it was performed within 14 days following any hospital discharge or (2) the patient had used anti-IL-6R treatment in the preceding 90 days (because tocilizumab treatment may affect the MBDA score in a way that might confound CVD risk prediction) [36]; in these cases, the next MBDA test meeting the above requirements was used and the baseline period was anchored to that test. The follow-up period for ascertaining CVD outcomes (see below) began on the date of the first qualifying MBDA test. The follow-up period ended at the earliest of (1) a CVD outcome, (2) diagnosis of malignancy, (3) non-CVD death, or (4) the end of study (December 31, 2016).

### CVD outcome

The CVD outcome we used for the prognostic test was a composite, defined as the occurrence of hospitalized MI, stroke, or fatal CVD. This outcome definition is consistent with the outcome used in the guidelines of the ACC/AHA [9]. MI was defined as ICD-9 diagnosis code 410.x1 or ICD-10 diagnosis code I21.* from an inpatient hospitalization lasting ≥ 1 night or where the patient died. Stroke was identified using ICD-9 diagnosis codes 430.*, 431.*, 433.x1, 434.x1, 436.* or ICD-10 diagnosis codes I60.*, I61.*, I63.* or I67.89 from hospital discharge. This approach has been described previously [37–39]. Fatal CVD was identified using a validated algorithm that identifies fatal MIs and fatal strokes from Medicare data at a threshold yielding a positive predictive value > 80%, with greater accuracy than is obtained using hospital discharge diagnoses [40].

### Biomarkers and other predictors

#### MBDA score

All biomarker data in this study came from the MBDA test, which measures the serum concentrations of 12 biomarkers and uses an algorithm to produce a disease activity score on a scale of 1 to 100. The MBDA score has been validated against DAS28-CRP in patients treated with a variety of RA therapies, with AUROC values of 0.77 and 0.70 observed in seropositive and seronegative RA patients, respectively [30, 41]. The MBDA score is used to assess and monitor inflammatory disease activity in RA patients and is complementary to clinical assessment. It is a stronger predictor of risk for radiographic progression than DAS28-CRP [32, 33]. The MBDA score is not intended for the diagnosis of RA but rather is for use in assessing disease activity in patients with already-diagnosed RA. The MBDA score has been available for use in clinical practice in the US since 2010. Its cost has been covered in the US by Medicare since 2013 and is also covered by some private insurers.

The biomarkers in the MBDA test reflect the biology of RA and comprise cytokine-related proteins (IL-6, TNF-R1), acute phase reactants (CRP, serum amyloid A), an adhesion molecule (VCAM-1), a skeletal-related protein (bone glycoprotein 39 [YKL-40]), growth factors (EGF, VEGF-A), matrix metalloproteinases (MMP-1, MMP-3), and adipokines (leptin, resistin). All MBDA scores analyzed here were from tests that had been ordered by practitioners in the US as part of routine patient care. All MBDA testing was performed in a Clinical Laboratory Improvements Amendment-certified commercial laboratory in South San Francisco, CA (Crescendo Bioscience), where MBDA scores were calculated and stored with related data in a secure database.

Prior to and independently of the present study, an algorithm was developed and validated to adjust the MBDA score for the effects of age, sex, and leptin (as a surrogate for adiposity) [42]. This adjustment acts on the original MBDA score without affecting the individual contributions of the 12 biomarkers. Thus, the original MBDA score is calculated as previously, then adjusted to produce a score that, like the original score, has a scale of 1–100 and RA disease activity categories of low (< 30), moderate (30–44), and high (> 44) [30, 42]. The adjusted MBDA score has been in routine use since December 2017. Original MBDA scores were converted to adjusted MBDA scores for this study. In the remainder of this report, the term “MBDA score” means the adjusted MBDA score.

#### Variables considered for inclusion in model building

Variables considered for use in model building that came from the MBDA database included the MBDA score and the serum concentrations of its 12 component biomarkers. This approach was non-redundant because the algorithm for the MBDA score is a non-linear combination of its component biomarkers, which were neither selected nor weighted for CVD prediction [30, 41].

Demographic and clinical predictors were obtained from the Medicare database and were considered for inclusion in model building based upon their expected association with CVD risk, informed by subject matter expertise and the medical literature. Other considerations were face validity, data quality in the Medicare database, and feasibility of collecting a variable accurately in clinical practice. These predictors included age, sex, race, tobacco use (past or present), history of CVD other than MI or stroke, diagnoses of and medications for diabetes, hypertension and hyperlipidemia, RA medications as described above, glucocorticoids, and non-steroidal anti-inflammatory drugs. A diagnosis was counted as present if any of its diagnostic codes was found for the patient. Diagnostic codes for the candidate predictors, i.e., the subset of variables that were included in the final model-building exercise, and the prevalences of CVD-related conditions, appear in Supplemental Table 2.

Clinical measurements (e.g., blood pressure or lipid levels) were not available in either database and were not considered for inclusion in model building. Current use of CV-related medications (e.g., lipid-lowering therapies) and RA medications was initially considered and was evaluated as part of baseline data assessment. However, a decision was made to not include any medications as variables in model building for two reasons: (1) without being able to account for disease-related clinical measurements, the estimated effect of medications may be counterintuitive or inaccurate and (2) suboptimal medication adherence could result in meaningful misclassification of the CV risk associated with these treatments. Race was excluded because of uncertainties related to racial heterogeneity and the reporting of race.

### Statistical analysis

A principled, pre-specified approach to model building and selection was conducted that followed Transparent Reporting of a multivariable prediction model for Individual Prognosis Or Diagnosis (TRIPOD) guidelines [43]. First, the cohort was randomly split 2:1 into separate datasets for training and testing (i.e., internal validation).

Prior to model building, the independent association of the MBDA score with the CVD risk was evaluated in the training dataset with a multivariable analysis that included all non-biomarker candidate predictors [16]. Separately, the form of the relationship between MBDA score and CVD risk, on the logarithmic scale of hazard, was examined and found to be linear up to MBDA scores of approximately 60 and non-linear thereafter—a relationship that can be described with a hyperbolic tangent function (see below), which is commonly used in other fields, e.g., in models of neural networks [44].

#### Training: evaluation of variables and model building

Model development was conducted in the training dataset, to achieve the goal of estimating individual risk for the composite CVD outcome as a function of the candidate predictors. Individual biomarker concentrations in ng/ml were natural log transformed. MBDA scores (integers on a scale of 1 to 100) were hyperbolic tangent-transformed, as *f*(*x*) = tanh(*a* ∗ *x*), where *a* is a constant parameter that was based on maximum likelihood estimation and updated in each step of model building. Age in years was treated as a continuous variable. A separate age-squared term was initially included to account for possible nonlinearity between age and the composite CVD outcome, but it added no additional value to model building and was dropped. Other candidate predictors were treated as binary variables.

Association with 3-year CVD risk was assessed for each candidate variable with a hazard ratio (HR) and determined by univariable analysis in the training dataset. A 3-year time frame was chosen based on the availability of MBDA biomarker data from testing performed as part of routine care. Model building used Cox proportional hazards regression with backward elimination in the training dataset. In the first step, a model was fit by including every candidate predictor variable; in each subsequent step, the least significant variable (i.e., with the highest *p* value) was removed, and the model was refit with the remaining variables. This process was repeated until all remaining variables had *p* < 0.05.

#### Clinical models developed for comparison

Four prespecified models for predicting CVD risk were built in the training dataset for comparison with the MBDA-based model: (1) age + sex, (2) age + sex + CRP, (3) a clinical model (age + sex + tobacco use + diabetes + hypertension + history of CVD [excluding MI and stroke]), and (4) the clinical model + CRP. These models were chosen for the availability of their variables in routine clinical practice and in our linked database.

#### Derivation of categories of 3-year risk for CVD events

The thresholds for 3-year CVD risk categories that would be equivalent to the thresholds for 10-year risk categories of other CVD risk prediction equations were derived in a cohort with 10 years of longitudinal data. To create a dataset in which CVD event rates at 3 and 10 years could be bridged, a cohort of 533,139 Medicare RA patients with data available from 2006 to 2016 was selected with the same requirements as for the main cohort of this study but without requiring MBDA testing. An age + sex model was developed in this cohort to establish 10-year rates of CVD events, and 3-year cutpoints corresponding to the 10-year ACC/AHA risk thresholds of 5% (± 0.1%), 7.5% (± 0.1%), and 20% (± 0.1%) [11] were obtained by bootstrapping. The derived cutpoints were 1.3%, 1.8%, and 5.2%, defining 3-year CVD risk categories of low (0 to < 1.3%), borderline (≥ 1.3 to < 1.8%), intermediate (≥ 1.8 to < 5.2%), and high (≥ 5.2%) risk.

#### Internal validation

The primary analysis for establishing internal validation was to estimate the risk of a composite CVD event at 3 years (i.e., the probability of a patient having an MI, a stroke, or CVD death in the next 3 years), by using the MBDA-based CVD risk score as the only variable in a Cox proportional hazard regression model. HR (with 95% confidence interval [CI]; *p* value by partial likelihood ratio test [LRT]) was determined for the MBDA-based CVD risk score [45–47]. A risk curve was constructed to illustrate this relationship, using methods described in Supplementary Text. These and all other validation analyses were performed in the validation dataset.

To assess accuracy of the MBDA-based CVD risk score, a secondary analysis for internal validation examined goodness of fit with plots that compared observed risk (based on Kaplan-Meier estimates with 95% CI) with predicted risk across CVD event-based deciles. *P* values were determined using the Greenwood-Nam-D’Agostino test [48], with higher (i.e., non-significant) *p* values indicating better fit. Goodness of fit was also assessed among patient subgroups, based on age, sex, diagnosis of diabetes, hypertension, tobacco use (past or present), and hyperlipidemia, as well as history of CVD, statin use, oral glucocorticoid use, initiation or change of a biologic agent during follow-up, and MBDA score category. Bonferroni correction was used to adjust for multiple testing. CVD event quintiles, rather than deciles, were used for patient subgroups with fewer than 110 CVD events to avoid data sparsity. In addition, Kaplan-Meier plots of CVD event-free status over time were constructed for patients grouped into CVD risk categories by the MBDA-based CVD risk score, using the Mantel-Haenszel test [45, 46].

Validation included comparisons of the predictive abilities of the MBDA-based CVD risk score and four clinical models described above. HR (95% CI) and *p* value (using the partial LRT) were calculated from Cox proportional hazards models in single-score (i.e., univariable) analyses of the MBDA-based CVD risk score and each of the four clinical models. To determine the incremental contribution of the MBDA-based model to each clinical model for predicting CVD risk (and vice versa), change in model deviance was determined using the likelihood ratio statistic in sequential (i.e., bivariable) analyses for each model pair.

The MBDA-based CVD risk model was also compared to the four clinical models with reclassification tables and the Net Reclassification Index (NRI) [49, 50]. The five models were each evaluated for discrimination based on the C-index (similar to AUROC) for predicting risk at 3 years, with times weighted by the square inverse of the censoring distribution [51].

#### Statistical software

SAS 9.4 was used for data preparation. R version 3.4 and R packages survival, nricens, and pec were used for evaluating model performance, calculating NRIs and C-indices, and generating plots [52].

## Results

### Cohort selection

30,751 RA patients with 904 CVD events (480 MI, 362 stroke, 62 CVD death) were eligible for the total cohort (Supplemental Table 1). Total follow-up from the index date was 56,684 patient-years (PY) with median (interquartile range [IQR]) follow-up duration of 1.7 (0.8–2.7) years. The overall CVD event rate (95% CI) was 15.9 (14.9–17.0) events per 1000 PY.

At baseline, the mean age was 69 years, 23% of patients were under age 65 years, 18% were men, and 8% were Black (Table 1). The prevalence of CVD-related comorbidities, such as diabetes (40%) and hypertension (79%), was high. Statin use was found in 42%. Sixty percent of patients were receiving methotrexate, 33% a TNF inhibitor (TNFi), and 15% a non-TNFi biologic. Median (IQR) CRP value was 4.5 (1.6–12.0) mg/L (or 1.5 [0.5–2.5] μg/ml natural log transformed). Median (IQR) MBDA score was 40 [32–49], which is in the moderate MBDA category (range, 30–44) (Table 1).

**Table 1:** Patient characteristics at baseline*

Predictors	Complete cohort *N* = 30,751	Patients with CVD event *N* = 904	Patients with no CVD event *N* = 29,847
**Age, mean (SD)**	68.8 (9.6)	72.7 (9.3)	68.6 (9.6)
**Age group, %**			
< 65 years	23.4	14.6	23.6
65–74 years	50.9	42.8	51.1
> 74 years	25.8	42.6	25.3
**Male, %**	18.2	23.0	18.1
**Black race, %**	8.4	6.9	8.4
Comorbidities*, %			
Diabetes	39.8	47.7	39.5
History of CVD	37.1	55.0	36.5
Hyperlipidemia	75.4	81.5	75.2
Hypertension	78.7	88.9	78.4
Obesity	12.1	8.7	12.2
Tobacco use (past or current)	24.5	27.7	24.3
**Medications, %**			
ACEI	25.9	32.4	25.7
ARB	22.0	24.2	22.0
Beta-blockers	34.4	48.6	34.0
Statins	42.4	45.1	42.4
**RA medications, %**			
Methotrexate	59.8	58.4	59.9
Other csDMARDs	44.7	44.5	44.7
TNFi biologics	32.8	29.5	32.9
Non-TNFi biologics	14.8	15.8	14.8
Abatacept	9.9	11.3	9.9
Rituximab	3.8	4.1	3.8
Tocilizimab	1.9	1.4	2.0
Tofacitinib	1.7	< 1.2	1.7
Oral glucocorticoids	57.5	62.7	57.4
NSAIDs	48.0	45.0	48.1
**Biomarkers†, median [IQR]**			
CRP (ug/ml)	1.5 [0.5, 2.5]	1.9 [0.9, 2.9]	1.5 [0.5, 2.5]
EGF (pg/ml)	4.5 [3.7, 5.1]	4.4 [3.7, 5.0]	4.5 [3.7, 5.1]
IL-6 (pg/ml)	2.6 [2.1, 3.3]	2.8 [2.3, 3.6]	2.6 [2.1, 3.3]
Leptin (ng/ml)	3.2 [2.3, 3.9]	3.0 [2.1, 3.7]	3.2 [2.3, 3.9]
MMP-1 (ng/ml)	1.9 [1.4, 2.4]	1.9 [1.4, 2.4]	1.9 [1.4, 2.4]
MMP-3 (ng/ml)	3.3 [2.8, 3.8]	3.5 [3.0, 4.1]	3.3 [2.8, 3.8]
Resistin (ng/ml)	2.1 [1.8, 2.4]	2.1 [1.9, 2.5]	2.1 [1.8, 2.4]
SAA (ug/ml)	1.0 [0.3, 1.9]	1.3 [0.5, 2.5]	1.0 [0.3, 1.9]
TNF-R1 (ng/ml)	0.6 [0.3, 0.8]	0.8 [0.5, 1.1]	0.6 [0.3, 0.8]
VCAM1 (ug/ml)	− 0.4 [− 0.5, − 0.2]	− 0.3 [− 0.5, − 0.1]	− 0.4 [− 0.5, − 0.2]
VEGF (pg/ml	5.5 [5.1, 5.9]	5.6 [5.2, 6.0]	5.5 [5.1, 5.9]
YKL-40 (ng/ml)	4.7 [4.2, 5.3]	5.1 [4.5, 5.6]	4.7 [4.2, 5.3]
**MBDA score, median [IQR]**	40.0 [32.0, 49.0]	44.0 [36.0, 54.0]	40.0 [32.0, 48.0]

*Based on diagnostic codes and administration and fill information in the baseline period (see Methods)

†All biomarker concentrations were from the MBDA test at the end of the baseline period and are natural log transformed. MBDA score is the adjusted score

CVD event is myocardial infarction, stroke, or CV death in 3 years from baseline

*ACEI* angiotensin converting enzyme inhibitor, *ARB* angiotensin receptor blocker, *csDMARD* conventional synthetic DMARD, *CVD* cardiovascular disease, *IQR* interquartile range, *MBDA* multi-biomarker disease activity (adjusted), *NSAID* non-steroidal anti-inflammatory drug, *SD* standard deviation, *TNFi* tumor necrosis factor inhibitor

### Confirming the MBDA score as an independent predictor of CVD risk

In the training dataset (*N* = 20,476 patients with 611 CVD events), the MBDA score, untransformed, was significantly prognostic of CVD events in a multivariable analysis with age, sex, diabetes, hypertension, tobacco use, CVD history, and hyperlipidemia, but with no individual biomarker variables (HR = 1.023; 95% CI 1.017–1.029).

### Training of the MBDA-based model

In univariable analyses in the training dataset, all candidate predictors except EGF and MMP-1 were individually predictive of CVD events (Table 2). In the final MBDA-based model, derived from backward elimination, the variables of age, diabetes, history of CVD, hypertension, tobacco use, MBDA score, and three biomarkers (leptin, MMP-3, TNF-R1) were significant predictors in multivariable analyses; sex, hyperlipidemia, and nine biomarkers were not. HRs were significantly > 1.0 for all predictor variables in the final MBDA-based model except leptin, for which HR was 0.84, indicating a negative relationship between leptin concentration and CVD risk (Table 2).

**Table 2:** Hazard ratios (HR) of predictor variables used in CVD risk models (training dataset, *N* = 20,476)

Predictors	Univariable models		Multivariable models					
	HR (CI)	***p*** value	Age + Sex HR (CI)	Age + Sex + CRP HR (CI)	Clinical HR (CI)	Clinical + CRP HR (CI)	Final MBDA-based model	
							HR (CI)	***p*** value
**Age**	1.05 (1.04–1.06)	3.45 × 10^−24^	1.05 (1.04–1.06)	1.05 (1.04–1.06)	1.04 (1.03–1.05)	1.04 (1.03–1.05)	1.03 (1.02–1.04)	6.68 × 10^−11^
**Male**	1.39 (1.15–1.68)	9.29 × 10^−4^	1.43 (1.19–1.73)	1.43 (1.19–1.73)	1.31 (1.08–1.59)	1.32 (1.09–1.60)	–	–
Comorbidities*								
Diabetes	1.49 (1.27–1.74)	1.08 × 10^−6^	–	–	1.31 (1.11–1.54)	1.29 (1.10–1.52)	1.31 (1.11–1.55)	0.0012
History of CVD	2.01 (1.71–2.35)	1.08 × 10^−17^	–	–	1.47 (1.24–1.74)	1.44 (1.21–1.71)	1.40 (1.18–1.66)	9.85 × 10^−5^
Hyperlipidemia	1.36 (1.11–1.66)	0.0023	–	–	–	–	–	–
Hypertension	2.52 (1.84–3.46)	5.49 × 10^−11^	–	–	1.35 (1.04–1.75)	1.30 (1.00–1.69)	1.31 (1.01–1.71)	0.0405
Tobacco use	1.38 (1.16–1.65)	4.55 × 10^−4^	–	–	1.42 (1.19–1.70)	1.35 (1.12–1.61)	1.31 (1.09–1.57)	0.0044
**Molecular**								
MBDA score	1.02 (1.02–1.03)	4.98 × 10^−14^	–	–	–	–	–	–
Tanh-MBDA^‡^	15.35 (7.17–32.87)	3.70 × 10^− 14^	–	–	–	–	4.99 (2.24–11.13)	4.22 × 10^−5^
CRP^†^, mg/L	1.20 (1.13–1.26)	7.04 × 10^−11^	–	1.22 (1.16–1.29)	–	1.20 (1.13–1.26)	–	–
EGF^†^, ng/mL	0.92 (0.85–1.00)	0.0650	–	–	–	–	–	–
IL-6^†^, ng/mL	1.29 (1.20–1.38)	1.92 × 10^−11^	–	–	–	–	–	–
Leptin^†^, ng/mL	0.87 (0.81–0.93)	7.93 × 10^−5^	–	–	–	–	0.84 (0.79–0.90)	2.99 × 10^−6^
MMP-1^†^, ng/mL	1.07 (0.96–1.19)	0.2497	–	–	–	–	–	–
MMP-3^†^, ng/mL	1.49 (1.35–1.65)	4.43 × 10^−14^	–	–	–	–	1.16 (1.03–1.30)	0.0139
Resistin^†^, ng/mL	1.58 (1.32–1.90)	7.32 × 10^−7^	–	–	–	–	–	–
SAA^†^, ng/mL	1.19 (1.13–1.26)	9.38 × 10^−10^	–	–	–	–	–	–
TNF-R1^†^, ng/mL	2.83 (2.39–3.35)	8.32 × 10^−29^	–	–	–	–	1.77 (1.43–2.19)	3.33 × 10^−11^
VCAM-1^†^, ng/mL	3.04 (2.36–3.91)	1.13 × 10^−16^	–	–	–	–	–	–
VEGF^†^, ng/mL	1.35 (1.19–1.54)	5.20 × 10^−6^	–	–	–	–	–	–
YKL-40^†^, ng/mL	1.60 (1.46–1.76)	1.30 × 10^−21^	–	–	–	–	–	–

These predictors comprise the complete list of predictors tested by backwards elimination to build the final MBDA-based model; also shown is the adjusted MBDA score, untransformed. *P* values by the likelihood ratio test

*Based on diagnostic codes during the baseline period (see the “Methods” section)

^†^Natural log transformed

^‡^Hyperbolic tangent transformed (tanh [*a* × MBDA Score [adjusted]], where *a* = 1/33.0807)

Clinical model includes age + sex + tobacco use + diabetes + hypertension + history of CVD (excluding MI and stroke)

*CI* 95% confidence interval, *CVD* cardiovascular disease, *MBDA* multi-biomarker disease activity

The equation for the final MBDA-based CVD risk score was:

$$ 0.0314\times \boldsymbol{age}\kern0.3em +\kern0.3em 0.2691\times \boldsymbol{tobacco}\;\boldsymbol{use}+\kern0.3em 0.2732\times \boldsymbol{diabetes}+0.2694\times \boldsymbol{hypertension}+0.3378\times \boldsymbol{history}\kern0.17em \boldsymbol{of}\;\boldsymbol{CVD}-0.1711\times \mathrm{In}\left(\boldsymbol{Leptin}\right)+0.1454\times \ln \left(\boldsymbol{MMP}\mathbf{3}\right)+0.5724\times \ln \left(\boldsymbol{TNFR1}\right)+1.6076\times \tanh \left(\boldsymbol{MBDA}\kern0.17em \boldsymbol{score}/33.0807\right), $$

where the age is in years, clinical variables are scored as 1 when present and zero when absent, Leptin, MMP-3, and TNF-R1 represent serum concentrations in ng/mL, the term “ln” means natural logarithm, and “tanh” means hyperbolic tangent transformation. The output of this algorithm is the MBDA-based CVD risk score. This score is used in a separate formula to calculate the predicted 3-year risk for a CVD event as a percentage value (see Supplemental Text).

In the four multivariable clinical models that were generated for comparison—i.e., an age + sex model and an age + sex + diabetes + hypertension + history of CVD + tobacco use model, each one with and without CRP—all variables in each model were significant CVD predictors (Table 2).

### Internal validation of the MBDA-based model

**Fig. 1:**
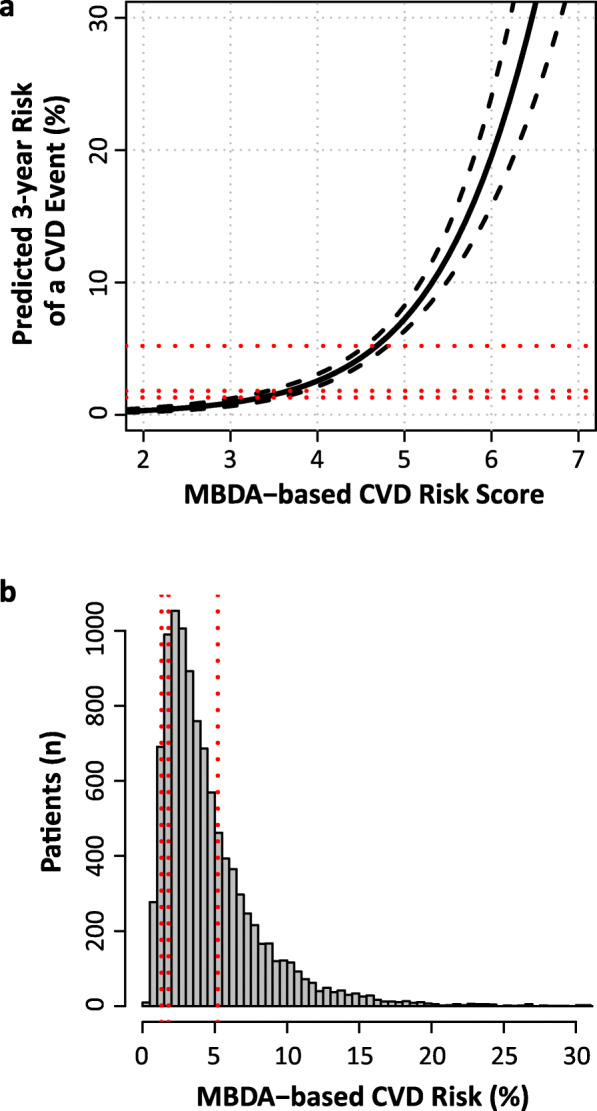
Characterization of the MBDA CVD risk score in the validation dataset (*N* = 10,275). **a** Relationship between MBDA-based CVD risk score and predicted 3-year risk of a CVD event, with 95% confidence interval. **b** Distribution of predicted 3-year risks. Dotted lines, horizontal in **a** and vertical in **b**, indicate thresholds at 1.3%, 1.8%, and 5.2% separating the categories of low, borderline, intermediate, and high risk, which contained 9.4%, 10.2%, 52.2%, and 28.2% of patients, respectively. CVD event is myocardial infarction, stroke, or CVD death. CVD cardiovascular disease, MBDA multi-biomarker disease activity

The MBDA-based CVD risk score was a strong predictor of 3-year risk for a CVD event in the validation dataset (*N* = 10,275 patients with 293 CVD events), with an HR (95% CI) of 2.89 (2.46–3.41, *p* = 4.67 × 10^− 38^). The relationship between the MBDA-based CVD risk score and predicted 3-year CVD risk is shown in Fig. 1a. The proportions of patients in the low, borderline, intermediate, and high categories of predicted 3-year CVD risk in the validation dataset were 9.4%, 10.2%, 52.2%, and 28.2%, respectively (Fig. 1b).

#### Assessment of accuracy with goodness of fit

**Fig. 2:**
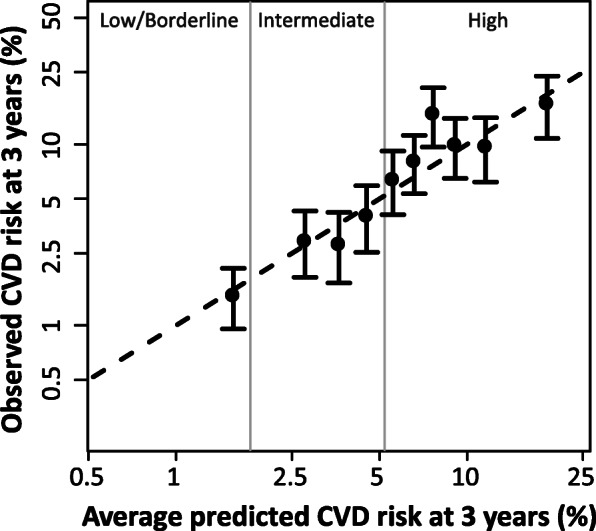
Goodness of fit: Predicted CVD risk versus observed 3-year CVD event rates. The observed 3-year CVD event rate was determined for each event-based decile and is shown vs. the average predicted 3-year risk in each decile. Analysis used the validation dataset (*N* = 10,275). Observed event rates were determined as Kaplan-Meier (95% log-log CI) estimates. *P* = 0.39 by the Greenwood-Nam-D’Agostino test, indicating good fit. CVD event is myocardial infarction, stroke, or CV death. 3-year CVD risk categories (low, borderline, intermediate, high) were derived from the 10-year risk categories of the 2018 Guidelines of the American College of Cardiology/American Heart Association [8]. Threshold between low and borderline risk categories is 1.3% (not shown). CI confidence interval, CVD cardiovascular disease, MBDA multi-biomarker disease activity

The 3-year CVD risk predictions made by the MBDA-based model were similar to the observed CVD event rates across deciles based on observed CVD events (Fig. 2). The goodness of fit test statistic indicated good fit (*p* = 0.39). The confidence intervals for observed risk contained the average predicted risk for all but one decile group. Overall, the mean predicted 3-year CVD risk in the validation dataset was 4.5%, compared with the observed 3-year CVD risk of 4.4%. Subanalyses showed that the MBDA-based model performed well in subgroups of interest: males and females, with/without diagnosis of diabetes, with/without diagnosis of hypertension, with/without tobacco use, with/without history of CVD, with/without hyperlipidemia, taking/not taking statins, < 65 years old, < 75 years old, and patients who had or had not used oral glucocorticoids in the baseline period, or initiated or changed a biologic drug during the follow-up period, or had low, moderate, or high disease activity (MBDA score) (Supplemental Fig. 1).

#### Loss of CVD outcome-free status by category of predicted risk

**Fig. 3:**
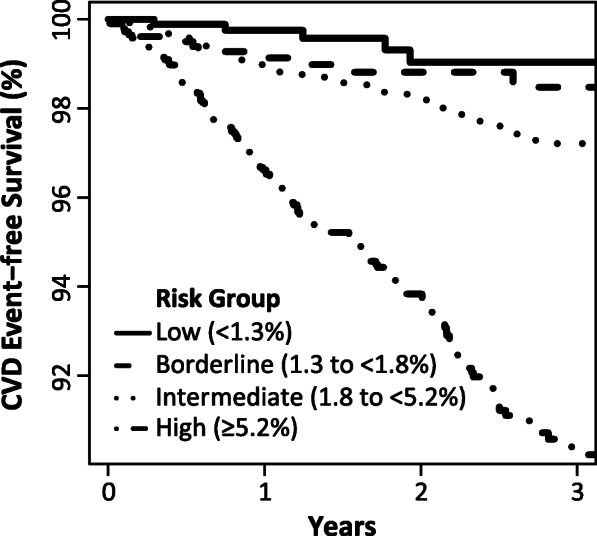
Kaplan-Meier plot of CVD event-free survival. Occurrence of CVD events by Kaplan-Meier survival analysis is shown for patients in the validation dataset (*N* = 10,275) grouped by a 3-year CVD risk category predicted by the MBDA-based CVD risk score at baseline. *P* = 1.7 × 10^−32^ by the Mantel-Haenszel test. CVD event is myocardial infarction, stroke, or CVD death. See Fig. 2 for explanation of CVD risk categories. CVD cardiovascular disease, MBDA multi-biomarker disease activity

A Kaplan-Meier plot depicting loss of CVD outcome-free status in the validation dataset showed statistically significant separation of the low, borderline, intermediate and high predicted CVD risk groups over time (*p* = 1.7 × 10^−32^) (Fig. 3).

#### Model evaluation and comparison by likelihood test

**Fig. 4:**
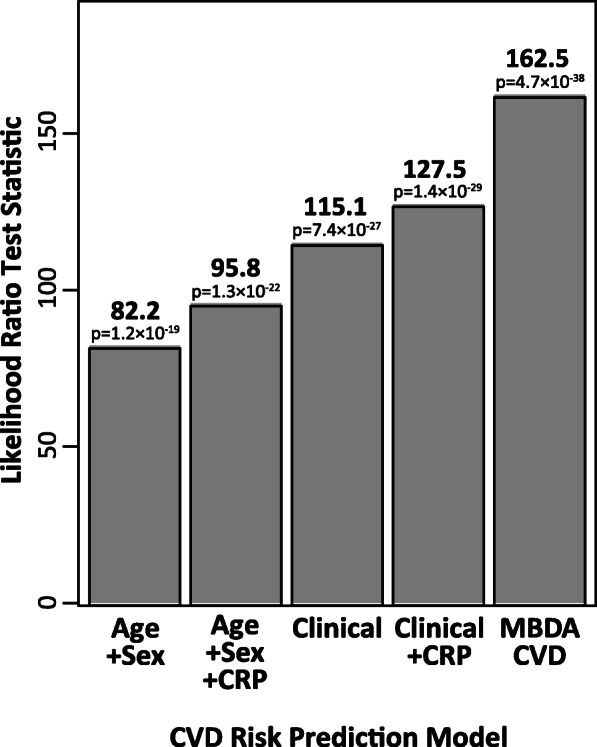
Contribution to CVD risk prediction by MBDA-based CVD risk score and clinical models. Likelihood ratio test statistics are shown for univariable (i.e., single-score) analyses of a CVD risk prediction by the MBDA-based CVD risk score and four comparison models, using the validation dataset (*N* = 10,275) (see also Table 3). *P* values are by the likelihood ratio test. The clinical model includes age, sex, tobacco use, diabetes, hypertension, and history of CVD. CRP C-reactive protein, CVD cardiovascular disease, MBDA multi-biomarker disease activity

When analyzed alone, each of the four clinical models made statistically significant contributions to the prediction of CVD risk in terms of the likelihood ratio, which represents how well the model fits the data (Fig. 4). However, these models made smaller contributions than the MBDA-based CVD risk score (Fig. 4). Moreover, the addition of these clinical models to the MBDA-based CVD risk score in paired analyses did not improve CVD risk prediction, as indicated by the respective increments in LRT statistic (0.4–3.0), which were small and non-significant (Table 3). In contrast, the MBDA-based CVD risk score provided additional information to improve the prediction of CVD risk when it was added to each clinical model, with the increments in LRT statistic being large (35.4–83.3) and statistically significant (all *p* < 3 × 10^− 9^) (Table 3).

**Table 3:** Contribution of MBDA-based CVD risk score and other models to prediction of 3-year CVD risk

CVD risk prediction score	Single-score analyses (univariable)			Sequential analyses of paired scores (bivariable)					
				A. Non-MBDA-based CVD Risk Score added to a base model of MBDA-based CVD Risk Score			B. MBDA-based CVD Risk Score added to a non-MBDA-based CVD Risk Score base model		
	HR (95% CI)	LRT statistic	***p*** value	HR (95% CI)	Increment in LRT statistic	***p*** value	HR (95% CI)	Increment in LRT statistic	***p*** value
**Age + sex**	**3.44** (2.62–4.53)	82.2	1.22 × 10^−19^	1.34 (0.96–1.86)	3.0	0.084	**2.62** (2.14–3.20)	83.3	7.15 × 10^−20^
**Age + sex + CRP**	**2.97** (2.38–3.70)	95.8	1.29 × 10^−22^	1.13 (0.84–1.54)	0.7	0.412	**2.71** (2.15–3.41)	67.4	2.20 × 10^− 16^
**Clinical**	**3.08** (2.50–3.80)	115.1	7.34 × 10^−27^	1.24 (0.89–1.71)	1.7	0.197	**2.56** (1.98–3.29)	49.0	2.51 × 10^−12^
**Clinical + CRP**	**2.94** (2.43–3.55)	127.5	1.44 × 10^−29^	1.12 (0.79–1.60)	0.4	0.526	**2.67** (1.96–3.63)	35.4	2.67 × 10^−9^
**MBDA-based CVD risk score**	**2.89** (2.46–3.41)	162.5	4.67 × 10^−38^	–	–	–	–	–	–

In single-score (univariable) analyses, each of the five risk scores derived from training was analyzed as a single independent variable for predicting risk for a CVD event in the validation dataset (*N* = 10,275). In sequential (bivariable) analyses of paired scores, the risk scores of the MBDA-based model and each non-MBDA-based risk model were evaluated as the only two variables used to predict CVD risk: (A) with the MBDA-based risk score as the base model and (B) with the non-MBDA-based CVD risk score as the base model. The increment in LRT statistic represents the extent to which a second CVD risk score adds to the CVD risk prediction ability of a first CVD risk score (i.e., the base model). Non-MBDA-based CVD risk score refers to the first four scores in the first column. *P* values by the likelihood ratio test. Statistically significant HR values are bolded. Clinical model includes age + sex + tobacco use + diabetes + hypertension + history of CVD (excluding MI and stroke)

*CRP* C-reactive protein, *CVD* cardiovascular disease, *HR* hazard ratio, *LRT* likelihood ratio test, *MBDA* multi-biomarker disease activity

#### Reclassification

Compared to the simplest of the clinical models, the age + sex model, the MBDA-based model reclassified the CVD risk for 42% of patients overall and as many as 75% of patients, depending on the age + sex model risk category (Table 4A). Compared to the most comprehensive clinical model, the clinical + CRP model, the MBDA-based model reclassified the CVD risk for 28% of patients overall and as many as 64% of patients, depending on the clinical + CRP model risk category (Table 4B). Reclassification results for the age + sex + CRP model and the clinical model (without CRP) were generally intermediate to those of the other two models (Supplemental Tables 3A and 3B).

**Table 4:** Reclassification of patients by the MBDA-based CVD risk score versus: A, age + sex model and B, clinical + CRP model

**A.**						
**CVD risk predicted by age + sex model**	**CVD risk predicted by MBDA-based CVD model**				*Observed cumulative incidence*	Total patients (*n*) within category of age + sex model and % reclassified
	Low (< 1.3%)	Borderline (1.3 to < 1.8%)	Intermediate (1.8 to < 5.2%)	High (≥ 5.2%)		
Low (< 1.3%)	2.6%	**0.9%**	**0.9%**	**< 0.1%**	*1.4%*	460 (42.6%)
Borderline (1.3 to < 1.8%)	**1.7%**	1.4%	**2.5%**	**0.2%**	*1.3%*	600 (75.3%)
Intermediate (1.8 to < 5.2%)	**4.9%**	**7.3%**	37.1%	**11.0%**	*3.7%*	6185 (38.4%)
High (≥ 5.2%)	**0.1%**	**0.6%**	**11.8%**	17.0%	*7.8%*	3030 (42.4%)
*Observed cumulative incidence*	*0.9%*	*1.7%*	*3.1%*	*9.9%*	–	–
**B.**						
**CVD risk predicted by clinical + CRP model**	**CVD risk predicted by MBDA-based CVD model**				*Observed cumulative incidence*	Total patients (*n*) within category of clinical + CRP model and % reclassified
	Low (< 1.3%)	Borderline (1.3 to < 1.8%)	Intermediate (1.8 to 5.2%)	High (≥ 5.2%)		
Low (< 1.3%)	4.4%	**1.2%**	**0.5%**	**0.0%**	*1.3%*	627 (28.2%)
Borderline (1.3 to < 1.8%)	**3.0%**	3.0%	**2.3%**	**< 0.1%**	*1.3%*	853 (64.4%)
Intermediate (1.8 to < 5.2%)	**2.0%**	**6.0%**	41.5%	**5.4%**	*3.1%*	5644 (24.4%)
High (≥ 5.2%)	**0**	**< 0.1%**	**7.9%**	22.7%	*9.3%*	3151 (26.0%)
*Observed cumulative incidence*	*0.9%*	*1.7%*	*3.1%*	*9.9%*	–	–

Values in the 16 cross-classification cells are percentages of the total validation dataset (*N* = 10,275). Boldfaced values represent patients who were reclassified, i.e., they were classified differently by the MBDA-based CVD model and the other model. Observed cumulative incidence values represent CVD event rates among patients in a row or column. Percentages of patients reclassified are of the total number of patients in that row. Clinical + CRP model includes age + sex + tobacco use + diabetes + hypertension + history of CVD (excluding MI and stroke) and CRP

*CVD* cardiovascular disease, *MBDA* multi-biomarker disease activity

NRI test statistics demonstrated that the MBDA-based model significantly improved classification versus all four clinical models, with NRI test statistics (95% CI) of 0.19 (0.10–0.27) versus the age + sex model, 0.16 (0.08–0.23) versus the age + sex + CRP model, 0.10 (0.04–0.17) versus the clinical model, and 0.07 (0.001–0.13) versus the clinical + CRP model.

#### Discrimination

The C-index (95% CI) for the prediction of CVD risk at 3 years by the MBDA-based CVD risk score in the validation dataset was 0.715 (0.683–0.747), which was numerically greater than the C-index for each clinical model. The difference was greatest versus the simplest clinical model and least versus the most comprehensive clinical model, with C-indices (95% CI) of 0.661 (0.628–0.695) for the age + sex model, 0.674 (0.642–0.707) for the age + sex + CRP model, 0.688 (0.656–0.721) for the clinical model, and 0.696 (0.664–0.729) for the clinical + CRP model.

#### Relationship between individual biomarkers and MBDA-based CVD risk score

**Fig. 5:**
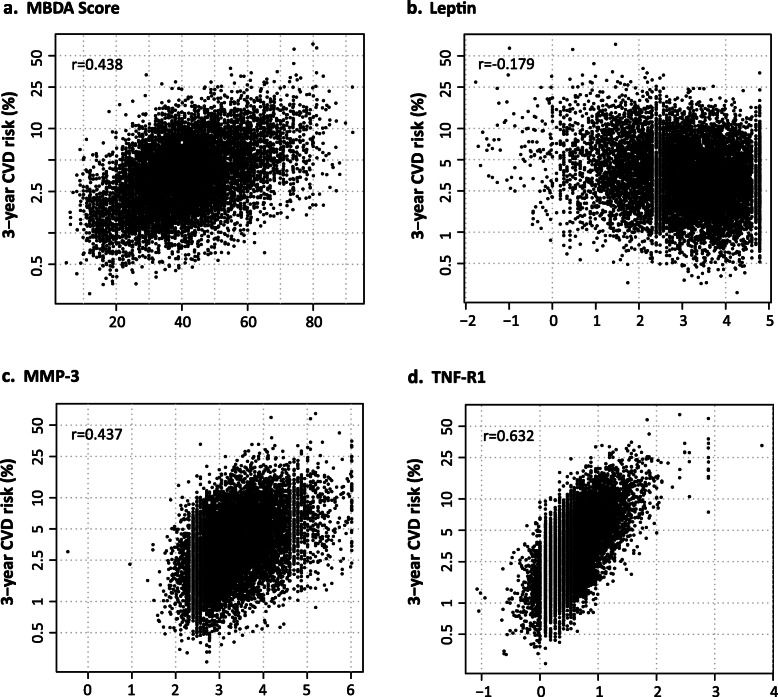
Relationship between predicted CVD risk and molecular variables. The predicted 3-year risk for a CVD event (myocardial infarction, stroke, or fatal CVD) is shown versus (**a**) the MBDA score and (**b**–**d**) serum concentrations (ng/ml, natural log transformed) of the three biomarker variables in the MBDA-based CVD risk score, using the validation dataset (*N* = 10,275). *R* values are Spearman correlation coefficients. CVD cardiovascular disease, MBDA multi-biomarker disease activity

Scatterplots derived from the validation dataset demonstrate the positive relationships between 3-year risk predicted by the MBDA-based CVD risk score and MBDA score (*r* = 0.438), MMP-3 (*r* = 0.437), and TNF-R1 (*r* = 0.632); and the negative relationship with leptin (*r* = − 0.179). For the MBDA score and for each biomarker, at most levels a range of CVD risks was observed, consistent with variation among the other variables of the MBDA-based CVD risk score (Fig. 5).

## Discussion

We have used a cohort of over 30,000 RA patients to derive and internally validate an MBDA-based CVD risk score for use in patients with RA. This score reflects the contribution of systemic inflammation to CVD risk by including the MBDA score and three individual biomarkers, while also incorporating age and four clinical risk factors. The MBDA-based risk score accurately predicted CVD risk in terms of goodness of fit analyses in the internal validation cohort and in clinically relevant subgroups, including patients who did or did not have prior CVD, who were already taking statins, or had different levels of RA disease activity. The MBDA-based risk score discriminated CVD risk better than clinical models, assigning some patients to higher or lower risk categories compared with clinical assessment alone.

This test is unique because it uses biomarker-based measurements to incorporate the contribution of RA inflammation to CVD risk in a more personalized way than by multiplying by a fixed value, such as 1.5 [6]. The MBDA score is a measure of RA disease activity that is also predictive of risk for radiographic progression. It was shown here and previously to be associated with the CVD risk [16], even though it was not originally developed for that purpose. MMP-3 and TNF-R1 were included in the final MBDA-based CVD risk score because in model building, they were positively associated with CVD risk independently of the MBDA score and other variables, which is consistent with previous reports of their role in cardiovascular risk [53–55].

The other individual biomarker in the CVD risk score was leptin. In our cohort, patients with a CVD event had less obesity and a numerically lower median leptin concentration than patients without a CVD event (Table 1). Leptin had a negative coefficient in the multivariable CVD risk prediction model. These results are consistent with evidence that leptin correlates strongly with body mass index (BMI) and that BMI has been negatively associated with CVD risk in RA patients [56], even though it is positively associated with CVD risk in the general population [57]. Our findings may reflect a contribution of RA inflammation to both weight loss and mortality, rather than a biologically protective effect of obesity [58]. They may also be a reflection of index case bias, which can lower the effect estimate for a risk factor, such as leptin, if it is associated with both the sequela of a disease and the disease itself, as with CVD events and RA [59]. IL-6, CRP, and other MBDA biomarkers were not included in the MBDA-based CVD risk score despite being individually associated with the CVD risk because none added significant information to leptin, MMP-3, TNF-R1, and the MBDA score for predicting CVD risk.

Clinical covariates that might have been expected in the final MBDA-based model, such as sex and hyperlipidemia, were associated with the CVD risk in univariable analyses but were not included because they made small incremental contributions to the multivariable model and did not survive the model building process. Sex was less significant as a univariable predictor of CVD risk than any of the variables that were included in the model (Table 2). It may have been excluded due to co-linearity with other variables, such as tobacco use, which is less common in women with RA than men with RA [4], and leptin, the levels of which tend to be greater in women [60]. It is unlikely that the MBDA score caused sex to be excluded from the model because adjustment of the MBDA score (for age, sex, and leptin) should have reduced its co-linearity with sex. The failure of hyperlipidemia to survive backward elimination may relate to it also having been a less significant univariable predictor of CVD risk than any of the predictors that survived. In addition, the “lipid paradox” [61] may make it difficult to interpret lipid values in RA patients, as they can be lower during active RA and increase with effective treatment. A practical consideration is that many RA patients have not had lipids tested recently, and co-management with primary care physicians may be needed to improve rates of screening for hyperlipidemia [62].

The cohort we used included patients with diabetes or a history of CVD and patients who were receiving statin treatment. Excluding such patients, as some CVD risk calculators do, would have greatly narrowed the utility of the score and reduced the power to see differences in the risk due to other variables. Instead, diabetes and a history of CVD were entered into model building as predictor variables and they were included in the score. Subanalyses demonstrated good fit between predicted and observed CVD events for patients with or without diabetes or a history of CVD. Statin use is not in the MBDA-based CVD risk score because we excluded drug-related variables from model building. However, the risk score demonstrated good fit in subanalyses of patients who were and were not receiving statins. The MBDA-based CVD risk score accounts for the level of inflammation, the treatment of which has potential to reduce CVD risk in RA patients [21–23]. The score may have utility for RA patients who are receiving statins because the statin dose may not yet have been optimized and because the non-statin treatment options for elevated CVD risk in RA patients may include DMARDs.

Other RA-specific CVD risk prediction models have been created. The expanded risk scored for CVD in RA (ERS-RA) was derived from a large RA cohort in the USA [19] and has been externally validated [28]. It quantifies RA disease activity categorically with the clinical disease activity index (CDAI) and also includes the Health Assessment Questionnaire (HAQ). A Trans-Atlantic Cardiovascular Risk Consortium for Rheumatoid Arthritis (ATACC-RA) developed two predictors that include serum lipid levels and account for RA disease activity with the 28-joint Disease Activity Score with erythrocyte sedimentation rate (DAS28-ESR) or HAQ, respectively [29]. The MBDA-based CVD risk score requires no clinical measurements and no laboratory data except results from the MBDA test. Rheumatologist preference among these predictors may depend on convenience and on which RA disease activity measures they use most routinely [63, 64]. CVD risk prediction for RA patients could be facilitated in a practical way if a risk score were to be automatically calculated—within an electronic medical record or, in the case of the MBDA-based CVD risk score, when the MBDA score is calculated by the testing laboratory—and provided to the ordering rheumatologist.

The large size of this study was made possible by linking administrative data from the Medicare database to a database of existing MBDA test results. The approach we used to capture CVD endpoint components in the Medicare database has a positive predictive value of approximately ≥ 93% for MI and 80–85% for stroke [37–39]. Fatal CVD events were identified using algorithms with positive predictive values ≥ 80% [40]. This study was restricted to patients ≥ 40 years old, to be aligned with the ACC/AHA guidelines [9]. A limitation of having used the Medicare cohort is that it contained predominantly older patients with high rates of CVD risk factors, and most of the 23% of patients < 65 years old were eligible for Medicare because they were disabled. In subanalyses of the patients who were < 65 years old and of patients who had or lacked each of the four clinical risk factors in the model, the MBDA-based CVD risk score had good fit with observed CVD events. In a previous report, CVD risk was relatively similar in younger disabled vs. younger non-disabled RA patients after accounting for the lower prevalence of CVD risk factors [65], suggesting that the MBDA-based CVD risk score may be applicable to patients < 65 years old who are not disabled. However, further validation of the CVD risk score in younger RA patients is needed.

Another limitation of our linked cohort is that clinical practice measurements, such as the blood pressure or lipid levels, were not available and the reasons for ordering MBDA tests were not known. Nevertheless, the MBDA-based CVD risk score demonstrated good fit with observed CVD events in patients with hypertension, hyperlipidemia, history of CVD or statin use, and in patients grouped by level of biomarker-based disease activity or according to whether a biologic DMARD treatment had been initiated or changed during follow-up. Because we lacked clinical measurements, the MBDA-based CVD risk score could not be compared with CVD risk predictors that require them, such as the ACC/AHA Pooled Cohort Equation or the Framingham Risk Score. As an alternative, the MBDA-based CVD risk score was compared to four clinical models of increasing complexity, from an age + sex model to a model that included age, sex, four traditional clinical risk factors available in the Medicare database, and CRP. The MBDA-based CVD risk score showed better fit than all four models, based on LRT. It also demonstrated statistically significantly better NRI and a numerically greater C-index. Because likelihood has been considered the most powerful means for comparing CVD risk prediction tests [66], and C-indices can fail to reflect meaningful incremental contributions of CVD-related biomarkers [67], these results suggest that the MBDA-based CVD risk score may be at least comparable to existing CVD risk calculators and potentially more practical for routine use. Direct comparison with other RA-specific calculators and general population CVD risk calculators adjusted for RA would be of interest.

The 3-year horizon used here for the composite CVD outcome reflects a constraint from the availability and uptake of the MBDA test for routine clinical practice in the US. Of more scientific relevance, however, is that RA is a dynamic disease and disease activity for many patients will fluctuate, such that a single measurement of disease activity may become less associated with true CVD risk over time. Thus, our shorter, 3-year time horizon may be preferable for predicting CVD risk in patients with RA, in that it is less subject to misclassification of RA disease activity than with the 10-year time horizon used by many existing CVD risk calculators. Indeed, the dynamic nature of RA disease activity and other factors that may be important to assessing CVD risk in RA patients is reflected in the ACC/AHA recommendation that, for adult patients with RA, “it can be useful to recheck lipid values and other major ASCVD (atherosclerotic CVD) risk factors 2 to 4 months *after the patient’s inflammatory disease has been controlled* [11].” Among all specialists, rheumatologists are likely in the best position to assess treatment response and systemic inflammatory burden in RA patients. The MBDA-based CVD risk score may assist rheumatologists by reminding them of the need for CVD risk management in RA patients—which some may wish to co-manage with a primary care physician or cardiologist—and of the unique role rheumatologists have in treating the inflammatory disease component of CVD risk [13].

## Conclusions

In conclusion, we have developed and internally validated an MBDA-based CVD risk score that predicts risk for MI, stroke, or fatal CVD in the next 3 years for RA patients. It is novel because it accounts for the contribution of RA inflammatory disease activity by including the MBDA score and three biomarkers that are independently associated with CVD. It performed better than prediction models that used only clinical data. The MBDA-based CVD risk prediction score provides rheumatologists with a feasible tool for assessing CVD risk to inform the management of traditional CVD risk factors and RA inflammation. Further validation with more extended time frames and more heterogeneous cohorts of RA patients will be helpful to assure its robustness as a prediction model.

## Supplementary information

**Additional file 1: Supplemental Figure 1.** Goodness of fit in patient subgroups (validation dataset, total *N*=10,275). **Supplemental Table 1.** Cohort Derivation. **Supplemental Table 2.** A, Diagnostic codes for candidate variables used to build the MBDA-based CVD risk score and B, Frequencies of CVD-related conditions comprising the History of CVD variable. **Supplemental Table 3.** Reclassification of patients based on CVD risk predicted by the MBDA-based CVD risk score versus: A, the Age + Sex + CRP model and B, the Clinical model. **Supplemental Text**. Conversion of the MBDA-based CVD risk score into 3-year percentage risk of a CVD event.

## Data Availability

The datasets used and/or analyzed during the current study are available from the corresponding author on reasonable request.

## Abbreviations

ACC/AHA: American College of Cardiology and American Heart Association
CDAI: Clinical Disease Activity Index
CI: Confidence interval
CRP: C-reactive protein
CVD: Cardiovascular disease
DAS28-CRP: Disease Activity Score in 28 joints with CRP
DAS28-ESR: Disease Activity Score in 28 joints with erythrocyte sedimentation rate
EULAR: European League Against Rheumatism
FRS: Framingham Risk Score
LRT: Likelihood ratio test
MBDA: Multi-biomarker disease activity
MI: Myocardial infarction
NRI: Net Reclassification Index
PY: Patient-years
RA: Rheumatoid arthritis
TNFi: TNF inhibitor
TRIPOD: Transparent Reporting of a multivariable prediction model for Individual Prognosis or Diagnosis
