# Supplementary material for: Derivation and internal validation of a multi-biomarker-based cardiovascular disease risk prediction score for rheumatoid arthritis patients
Source: Arthritis Res Ther. 2020 Dec 4;22:282. doi: 10.1186/s13075-020-02355-0 (PMC7718706; doi:10.1186/s13075-020-02355-0)
Supplement: Supplementary file 1 — Additional file 1: Supplemental Figure 1. Goodness of fit in patient subgroups (validation dataset, total N=10,275). Supplemental Table 1. Cohort Derivation. Supplemental Table 2. A, Diagnostic codes for candidate variables used to build the MBDA-based CVD risk score and B, Frequencies of CVD-related conditions comprising the History of CVD variable. Supplemental Table 3. Reclassification of patients based on CVD risk predicted by the MBDA-based CVD risk score versus: A, the Age + Sex + CRP model and B, the Clinical model. Supplemental Text. Conversion of the MBDA-based CVD risk score into 3-year percentage risk of a CVD event. [file 13075_2020_2355_MOESM1_ESM.pdf]

SUPPLEMENTARY MATERIALS

Supplemental Figure 1: Goodness of fit in patient subgroups (validation dataset, total N=10,275).

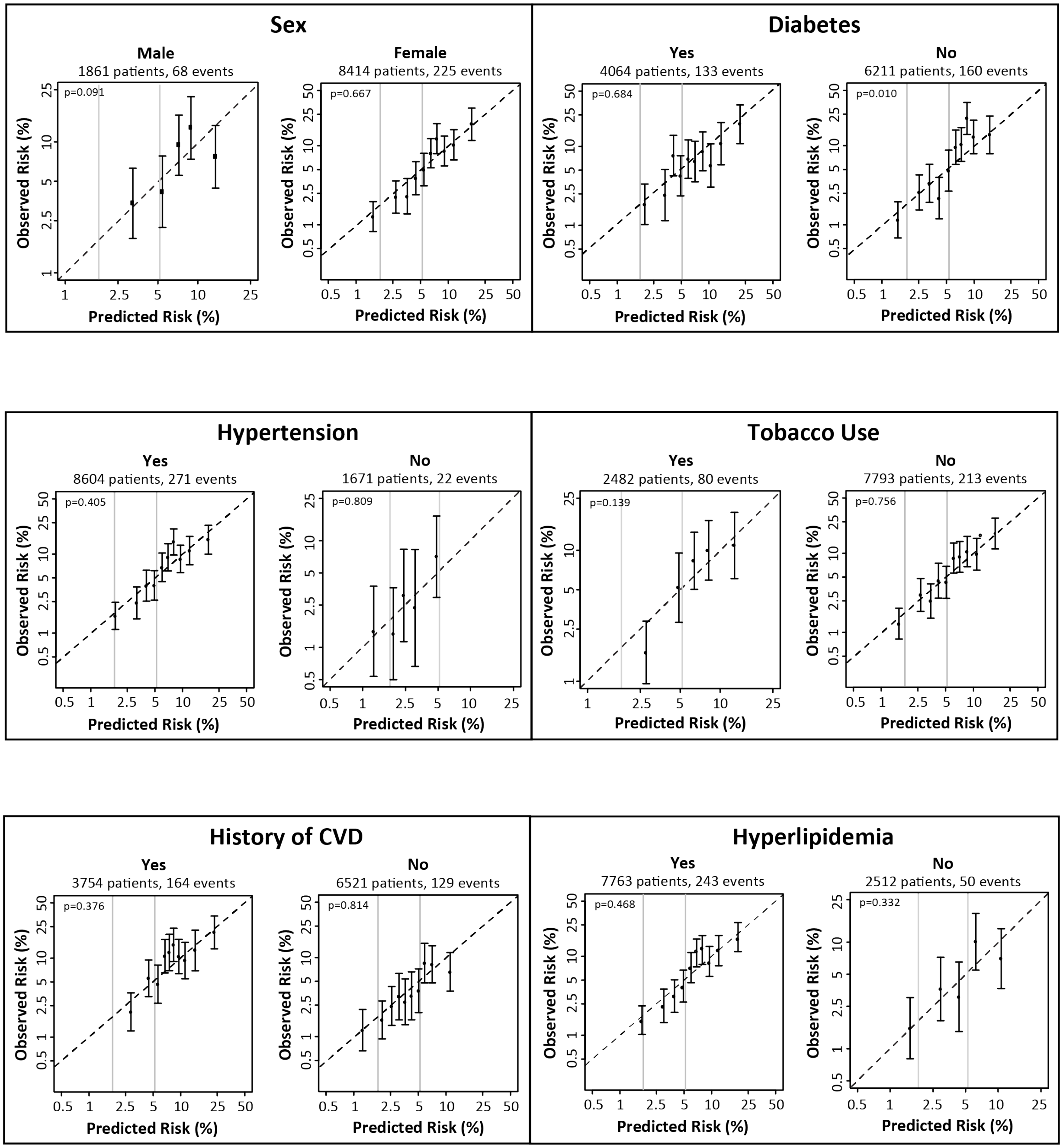

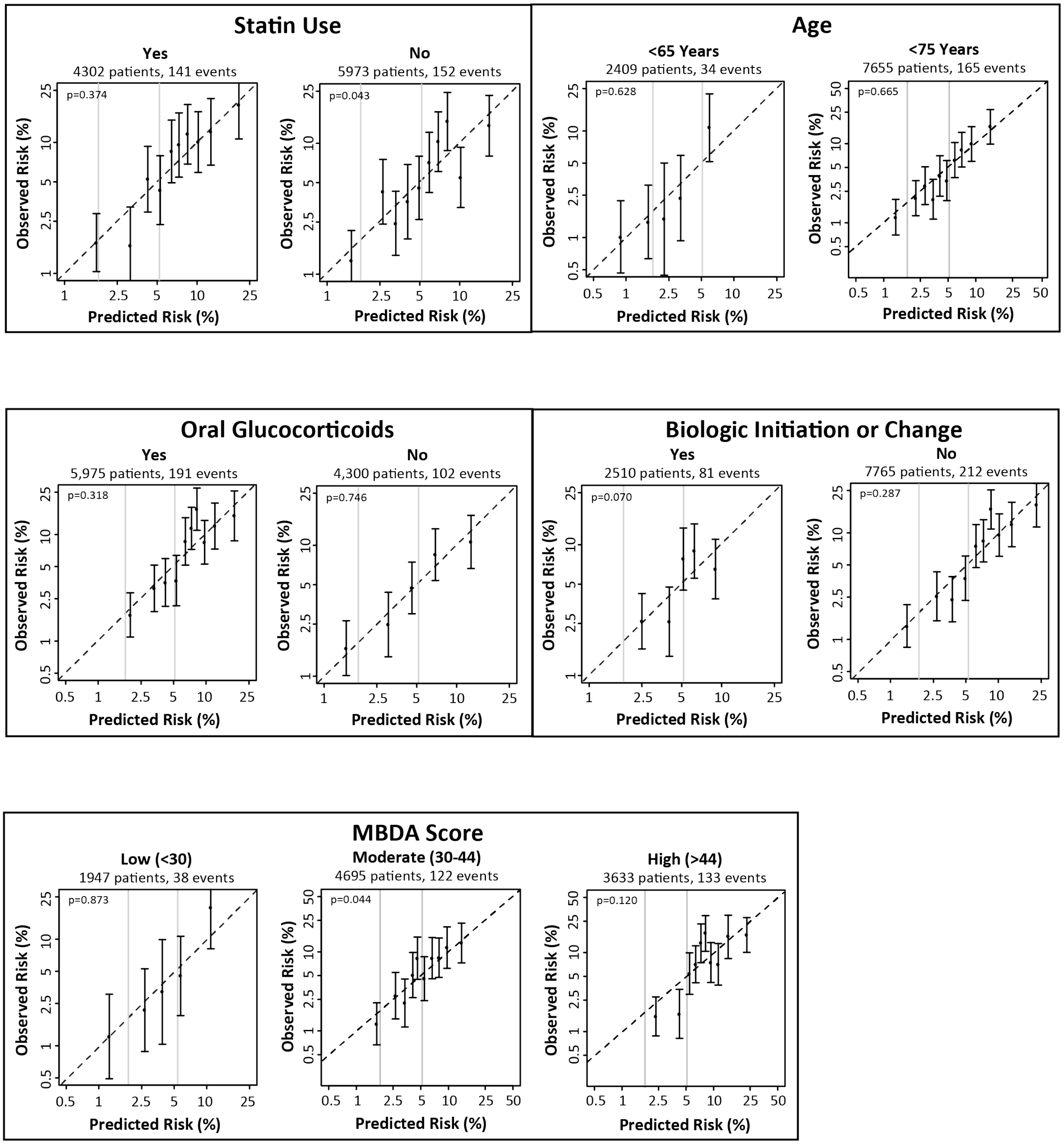

Kaplan Meier (95% log-log CI) estimate of observed 3-year CVD event rate was determined for each event-based decile in the indicated patient subgroup and is shown vs. the average predicted 3-year risk for each decile. Subgroups with <110 events were analyzed using event-based quintiles (see Methods). P-values are by the Greenwood-Nam-D'Agostino test. All p-values are >0.0022, which indicates at least adequate fit with Bonferroni correction for 23 sub-analyses (0.05/23). Predicted risk and observed risk are for a three-year period. Subgrouping was based on information from the baseline period except for the biologic initiation or change subgroups, which were based on post-baseline data. CVD event is myocardial infarction, stroke or CV death. Categories of 3-year CVD risk (low/borderline, intermediate and high) were derived from the 10-year risk categories of the 2018 Guidelines of the American College of Cardiology/American Heart Association (11) and are demarcated by vertical lines at 1.8% and 5.2%; threshold between low and borderline categories at 1.3% is not shown. CI, confidence interval, CVD, cardiovascular disease; MBDA, multi-biomarker disease activity.

Supplemental Table 1. Cohort Derivation

Selection Step	Number of patients	Number of observations
1. Vectra data received	150,887	359,302
2. Limited to 2010-2016	127,509	263,353
3. Linked to Medicare	87,492	188,902
4. Excluded un-valid tests (contaminated)*	86,039	182,451
5. Limited to 2011-2016	85,751	181,523
6. Required at least 365 days Part A, B, D, and not C enrollment at or before test date	47,500	93,931
7. Meet RA definition at or before test date	43,845	87,480
8. Keep first observation	43,845	43,845
9. Required DMARD use prior to test (using all available data)	41,490	41,490
10. Excluded subjects with prior diagnosis for malignancy, MI, stroke or old MI	31,322	31,322
11. Limited to age 40 or older	30,751	30,751
**Final Cohort**	**30,751**	30,751

*Reasons are not mutually exclusive: 1030 due to hospitalization within 14 days; 760 due to anti-IL-6R drug identified using National Drug Code within 90 days; 4814 due to anti-IL-6R drug identified using Healthcare Common Procedure Coding System code within 90 days.

Supplemental Table 2. A, Diagnostic codes for candidate variables used to build the MBDA-based CVD risk score and B, Frequencies of CVD-related conditions comprising the History of CVD variable.

A.

Diagnosis Code	ICD	Description	Clinical Variable in MBDA-based Model
249*	09	Secondary diabetes mellitus	Diabetes
250*	09	Diabetes mellitus	Diabetes
790.2x	09	Abnormal glucose	Diabetes
791.5x	09	Glycosuria	Diabetes
791.6x	09	Acetonuria	Diabetes
E08*	10	Diabetes mellitus due to underlying condition	Diabetes
E09*	10	Drug or chemical induced diabetes mellitus	Diabetes
E10*	10	Type 1 diabetes mellitus	Diabetes
E11*	10	Type 2 diabetes mellitus	Diabetes
E13*	10	Other specified diabetes mellitus	Diabetes
R73.0*	10	Abnormal glucose	Diabetes
R73.9*	10	Hyperglycemia, unspecified	Diabetes
V45.85	09	Insulin pump status	Diabetes
V53.91	09	Fitting and adjustment of insulin pump	Diabetes
V65.46	09	Encounter for insulin pump training	Diabetes
411*	09	Other acute and subacute forms of ischemic heart disease	History of CVD
413*	09	Angina pectoris	History of CVD
414*	09	Other forms of chronic ischemic heart disease	History of CVD
427.31	09	Atrial fibrillation	History of CVD
428*	09	Heart failure	History of CVD
432*	09	Other and unspecified intracranial hemorrhage	History of CVD
435*	09	Transient cerebral ischemia	History of CVD
G45.0*	10	Vertebro-basilar artery syndrome	History of CVD
G45.1*	10	Carotid artery syndrome (hemispheric)	History of CVD
G45.8*	10	Other transient cerebral ischemic attacks and related syndromes	History of CVD
G45.9*	10	Transient cerebral ischemic attack, unspecified	History of CVD
I20*	10	Angina pectoris	History of CVD
I24*	10	Other acute ischemic heart diseases	History of CVD
I25*	10	Chronic ischemic heart disease	History of CVD
I50*	10	Heart failure	History of CVD
I62*	10	Other and unspecified nontraumatic intracranial hemorrhage	History of CVD
440.2x	09	Atherosclerosis of native arteries of the extremities	History of CVD
440.31	09	Atherosclerosis of autologous vein bypass graft of the extremities	History of CVD
444.2	09	Embolism and thrombosis of arteries of the extremities	History of CVD

444.81	09	Arterial embolism and thrombosis of Iliac artery	History of CVD
I70.209*	10	Unspecified atherosclerosis of native arteries of extremities, unspecified extremity	History of CVD
I70.219*	10	Atherosclerosis of native arteries of extremities with intermittent claudication, unspecified extremity	History of CVD
I70.229*	10	Atherosclerosis of native arteries of extremities with rest pain, unspecified extremity	History of CVD
I70.25*	10	Atherosclerosis of native arteries of other extremities with ulceration	History of CVD
I70.269*	10	Atherosclerosis of native arteries of extremities with gangrene, unspecified extremity	History of CVD
I70.499*	10	Other atherosclerosis of autologous vein bypass graft(s) of the extremities, unspecified extremity	History of CVD
I74.2*	10	Embolism and thrombosis of arteries of the upper extremities	History of CVD
I74.3*	10	Embolism and thrombosis of arteries of the lower extremities	History of CVD
I74.5*	10	Embolism and thrombosis of iliac artery	History of CVD
441.3x	09	Abdominal aneurysm, ruptured	History of CVD
441.4x	09	Abdominal aneurysm without mention of rupture	History of CVD
441.5x	09	Aortic aneurysm of unspecified site, ruptured	History of CVD
441.6x	09	Thoracoabdominal aneurysm, ruptured	History of CVD
441.7x	09	Thoracoabdominal aneurysm, without mention of rupture	History of CVD
441.9x	09	Aortic aneurysm of unspecified site without mention of rupture	History of CVD
I71.3*	10	Abdominal aortic aneurysm, ruptured	History of CVD
I71.4*	10	Abdominal aortic aneurysm, without rupture	History of CVD
I71.5*	10	Thoracoabdominal aortic aneurysm, ruptured	History of CVD
I71.6*	10	Thoracoabdominal aortic aneurysm, without rupture	History of CVD
I71.8*	10	Aortic aneurysm of unspecified site, ruptured	History of CVD
I71.9*	10	Aortic aneurysm of unspecified site, without rupture	History of CVD
272.0x	09	Pure hypercholesterolemia	Hyperlipidemia
272.1x	09	Pure hyperglyceridemia	Hyperlipidemia
272.2x	09	Mixed hyperlipidemia	Hyperlipidemia
272.3x	09	Hyperchylomicronemia	Hyperlipidemia
272.4x	09	Other and unspecified hyperlipidemia	Hyperlipidemia
E78.0*	10	Pure hypercholesterolemia	Hyperlipidemia
E78.1*	10	Pure hyperglyceridemia	Hyperlipidemia
E78.2*	10	Mixed hyperlipidemia	Hyperlipidemia
E78.3*	10	Hyperchylomicronemia	Hyperlipidemia
E78.4*	10	Other hyperlipidemia	Hyperlipidemia
E78.5*	10	Hyperlipidemia, unspecified	Hyperlipidemia
401*	09	Essential hypertension	Hypertension
402*	09	Hypertensive heart disease	Hypertension
403*	09	Hypertensive chronic kidney disease	Hypertension

404*	09	Hypertensive heart and chronic kidney disease	Hypertension
405*	09	Secondary hypertension	Hypertension
437.2x	09	Hypertensive encephalopathy	Hypertension
I10*	10	Essential (primary) hypertension	Hypertension
I11*	10	Hypertensive heart disease	Hypertension
I12*	10	Hypertensive chronic kidney disease	Hypertension
I13*	10	Hypertensive heart and chronic kidney disease	Hypertension
I15*	10	Secondary hypertension	Hypertension
I16*	10	Hypertensive crisis	Hypertension
I67.4*	10	Hypertensive encephalopathy	Hypertension
N26.2*	10	Page kidney	Hypertension
305.1	09	Tobacco use disorder	Tobacco use
F172	10	Nicotine dependence	Tobacco use
V15.82	09	History of tobacco use	Tobacco use
Z87.891	10	Personal history of nicotine dependence	Tobacco use

B.

**CVD-related Condition***	**Frequency in total cohort (% of 30,751)**
Other acute and subacute forms of ischemic heart disease	2.68%
Angina pectoris	6.31%
Other forms of chronic ischemic heart disease	22.21%
Atrial fibrillation	9.43%
Heart failure	10.43%
Other and unspecified intracranial hemorrhage	0.24%
Transient cerebral ischemia	3.89%
Atherosclerosis	6.46%
Abdominal aneurysm, ruptured	1.66%

*Conditions are not mutually exclusive.

Supplemental Table 3. Reclassification of patients based on CVD risk predicted by the MBDA-based CVD risk score versus: A, the Age + Sex + CRP model and B, the Clinical model.

A.

**CVD risk predicted by Age+Sex + CRP Model**	**CVD risk predicted by MBDA-based CVD Model**				*Observed cumulative incidence*	Total patients (n) within category of Age+Sex+CRP model and % reclassified
	Low (<1.3%)	Borderline (1.3 to <1.8%)	Intermediate (1.8 to 5.2%)	High (≥5.2%)		
Low (<1.3%)	2.5%	0.8%	<0.1%	<0.1%	*2.8%*	416 (22.8 %)
Borderline (1.3 to <1.8%)	2.0%	1.5%	2.3%	0.1%	*1.3%*	614 (75.7%)
Intermediate (1.8 to <5.2%)	4.7%	7.7%	39.1%	9.2%	*3.1%*	6236 (35.6%)
High (≥5.2%)	<0.1%	0.2%	10.2%	18.9%	*8.9%*	3009 (35.6%)
Observed cumulative incidence	*0.9%*	*1.7%*	*3.1%*	*9.9%*		

B.

**CVD risk predicted by Clinical Model**	**CVD risk predicted by MBDA-based CVD Model**				*Observed cumulative incidence*	Total patients (n) within category of Clinical model and % reclassified
	Low (<1.3%)	Borderline (1.3 to <1.8%)	Intermediate (1.8 to 5.2%)	High (≥5.2%)		
Low (<1.3%)	3.8%	1.1%	0.6%	0.0%	*0.8%*	573 (31.2%)
Borderline (1.3 to <1.8%)	2.6%	2.4%	2.5%	<0.1%	*1.7%*	769 (68.3%)
Intermediate (1.8 to <5.2%)	2.9%	6.6%	39.4%	6.8%	*3.2%*	5721 (29.3%)
High (≥5.2%)	<0.1%	0.1%	9.8%	21.3%	*8.9%*	3212(31.8%)
Observed cumulative incidence	*0.9%*	*1.7%*	*3.1%*	*9.9%*		

Values in the 16 cross-classification cells are percentages of the total validation cohort (N=10,275). Observed cumulative incidence values represent CVD event rates among patients in a row or column. Percentages of patients reclassified are of the total number of patients in that row. Clinical model includes age + sex + tobacco use + diabetes + hypertension + history of CVD (excluding MI and stroke). CVD, cardiovascular disease. MBDA, multi-biomarker disease activity.

Supplemental Text

Risk score is converted into 3-year percentage risk of a CVD event using the following equation:

$$\text{Risk \%} = 100 \times (1 - A^{\exp[B \times \text{Risk Score}]}).$$

B is the maximum partial likelihood estimate of the log hazard ratio in the univariable Cox proportional hazards model which uses only the risk score to predict time to CVD event. *A* is the Breslow estimator of the 'baseline survival' (i.e. probability of CVD event-free survival after 3 years for a theoretical patient with a risk score equal to zero), calculated from the same univariable Cox model (1). See "Training of the MBDA-based model" section in Results for Risk Score formula.

1. Breslow NE. Discussion of the paper by D. R. Cox. J R Statist Soc B. 1972;34:216–217.
